# Short and Long Term Measures of Anxiety Exhibit Opposite Results

**DOI:** 10.1371/journal.pone.0048414

**Published:** 2012-10-31

**Authors:** Ehud Fonio, Yoav Benjamini, Ilan Golani

**Affiliations:** 1 Department of Neurobiology, Weizmann Institute of Science, Rehovot, Israel; 2 Department of Statistics and Operations Research, Tel Aviv University, Tel Aviv, Israel; 3 Department of Zoology, Tel Aviv University, Tel Aviv, Israel; University of Chicago, United States of America

## Abstract

Animal models of human diseases of the central nervous system, generalized anxiety disorder included, are essential for the study of the brain-behavior interface and obligatory for drug development; yet, these models fail to yield new insights and efficacious drugs. By increasing testing duration hundredfold and arena size tenfold, and comparing the behavior of the common animal model to that of wild mice, we raise concerns that chronic anxiety might have been measured at the wrong time, for the wrong duration, and in the wrong animal. Furthermore, the mice start the experimental session with a short period of transient adaptation to the novel environment (habituation period) and a long period reflecting the respective trait of the mice. Using common measures of anxiety reveals that mice exhibit opposite results during these periods suggesting that chronic anxiety should be measured during the post-habituation period. We recommend tools for measuring the transient period, and provide suggestions for characterizing the post habituation period.

## Introduction

Generalized Anxiety Disorder is a prevalent human disorder afflicting millions of people over the globe ]1],[2]. It is characterized by excessive worry about a variety of everyday problems for at least 6 months, thus different from anxiety in response to an acute challenge (DSM-IV-TR). The search for its cure, as in the study of other human diseases of the CNS including depression, autism and schizophrenia, is guided by the use of animal models [3],[4],[5],[6]. These animals are selected as models of the human disease because they are claimed to share essential features with it and can therefore be used for testing the therapeutic efficacy of candidate drugs [7],[8],[9],[10],[11],[12]. The predictive value of these animal models is being seriously questioned by scientists in both industry and academia [13],[14],[15], asserting that “animal models of human CNS diseases are notoriously unpredictive, failing in clinical trials with humans” [16]. They mark the development of better animal models as one of the most important scientific challenges that should accelerate the discovery of efficacious drugs [16].

Here we examine the predictive potential of a common animal model of Generalized Anxiety Disorder. To fulfill this potential, such model should exhibit chronic, persistent and stable anxious behavior [4],[8],[17],[18].

To examine whether these requirements are fulfilled by common models of chronic anxiety it is necessary to test the models in a setup that would expose their anxious behavior, and distinguish between transient states that are elicited by transitory stimuli whose influence fades out due to, e.g., habituation *versus* stable anxiety features reflecting the animal’s trait [8]. The apparatus and procedures used for testing anxiety include a polarity between sheltered area and intimidating locations involving high risk: wall versus center in the Open Field [19] (OF), home-cage versus exposed area in the Free Exploratory Paradigm [20] (FEP), closed versus open arms in the Elevated Plus Maze [21] (EPM) and dark versus bright chambers in the Light Dark test [22] (LD). The measures of anxiety estimate the conflict between tendencies to avoid and explore exposed spaces. The results obtained by them are, however, inconsistent and even contradictory [3],[4],[6],[9],[16],[17],[18],[23],[24],[25], and their interpretation is controversial [5],[8],[12]. Failure has been attributed to a multiplicity of causes, with arguments ranging from molecular biology to sociology of science (see Table S1). Here, while not going into the question of the validity of the common measures of anxiety, we embarrassingly find that before even attending to any of these causes, failure has been unavoidable given the much-too-short test durations, the wrong stage of exploration and the usage of wrong animal-models. We then indicate directions for remedies.

In our study of free exploration of a large novel arena performed by a mouse from a home shelter we found that behavior during the time intervals measured by researchers was radically different from the behavior observed later on [26],[27]. We therefore set out to measure anxiety for a much longer time, in a way that would shed light on these differences and offer a solution. By extending testing duration hundred folds we study both transient and enduring properties of the behavior. The anxiety measures we use in this study correspond to the classical ones. The animal of choice is the BALB/c inbred mouse strain commonly used as a model of anxiety [28],[29]. This strain is tested *vis-à-vis* its wild progenitor, *Mus musculus domesticus,* which provides a wildlife perspective to the domesticated strain [30].

A comparison of the behavior in the common forced assays and in our free assay is justified because i) all the above setups, whether forced or free, include a polarity between a sheltered and a non-sheltered area. ii) In all these tests, whether forced or free, there is a gradient between familiar and novel. As soon as the animal is placed in the all-novel environment in the forced open field test, symmetry is broken, be it in the forced setup in reference to the slightly more familiar place of entry that becomes the animal’s home base from which it performs excursions into the novel portion [31],[32], or, in reference to the much more familiar home cage, from which it also performs excursions into the novel arena. In all tests there is a gradual transition from novel to familiar; however, in the forced open field test the boundary between novel and familiar is ill-defined whereas in the DIEM assay (the Dimensionality Emergence assay consisting of a 250 cm diameter circular arena attached to a home cage through a doorway allowing deliberate passage between the 2 compartments, see *Experimental setup* in the Methods section in Information S1) it is well-defined [26]. iii) In all tests, whether forced or free, there is a process of habituation to novelty, which, by definition, implies state, not trait [8]. Whereas in our free setup the stage of extensive habituation is defined (see below), in forced exploration habituation has also been reported [9],[33],[6], but its boundary has been defined only in studies extending over a long enough period of time (e.g., [34]. The difference between forced and free exploration is, therefore of degree, not of kind, justifying a comparison. A main claim made in our study is that measuring chronic anxiety requires the chopping off of the initial, expected habituation stage, however small, both in forced and in free setups, focusing on the stable stage that follows it.

## Results

**Figure 1:**
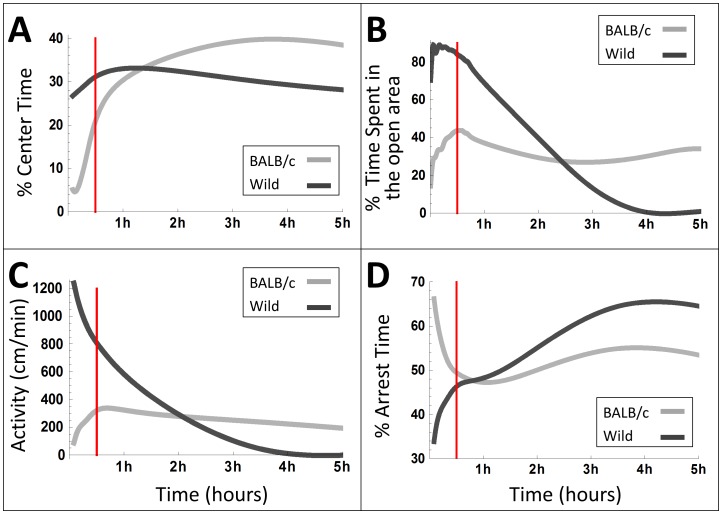
The dynamics of 4 classical measures of anxiety along the first 5 h in BALB/c (light gray) and wild (dark gray) mice. A: the percentage of time spent in the center of the arena, B: the percentage of time spent in the open area, C: Distance traveled (activity) per minute, D: percentage of time spent in arrest. Red vertical lines demarcate the end of the first half hour. Note that the trend, averaged over mice, shows a reversal in values of all 4 measures across the 5 h period and a consistent and large change across the first 1/2 h period, which is the maximal session length used in common studies of anxiety.

Figure 1 plots the dynamics of 4 classical measures of anxiety across a 5 h period. *1. % Center Time* is equivalent to the corresponding measure in the Open Field test, it measures the percentage of time spent in the center of the arena out of the total time spent in the arena (out of the home cage) *2. % of Time Spent in the open Area* is equivalent to the percentage of time spent in the open arms in the Elevated Plus Maze (EPM), it measures the percentage of time spent in the arena, outside the cage, out of the total testing duration of the given time interval. *3. Activity* presents distance traveled per minute, *and 4. % of Arrest* (freezing) *Time,* represents the percentage of time spent in arrest episodes (for the procedure of computing arrests and their correspondence to observer-defined freezing episodes see Methods in Information S1). All plots show a reversal that takes place somewhere between 0.75 h to 2.5 h after the beginning of the experiment, so that the values measured in the beginning and at the end of this period are radically different. Furthermore, it can be observed that over the first half hour, which is the maximal interval of data-collecting period in currently used tests of anxiolytic drugs (in most studies this period extends for an even shorter duration of only the first few minutes) bordered by the red vertical line in figure 1. Each of the measures undergoes in both strains a consistent and strong change. These time trends make the comparison across strains more difficult, as they add a substantial component to the variance of the average over the half hour variance that does not disappear even when the number of animals being measured is increased. At the beginning of the session the BALB/c mice score lower on the 4 measures, implying higher anxiety than the wild mice, whereas later on they score higher, implying lower estimated anxiety (for 3 additional variables that support these conclusions see Figure S1).

**Figure 2:**
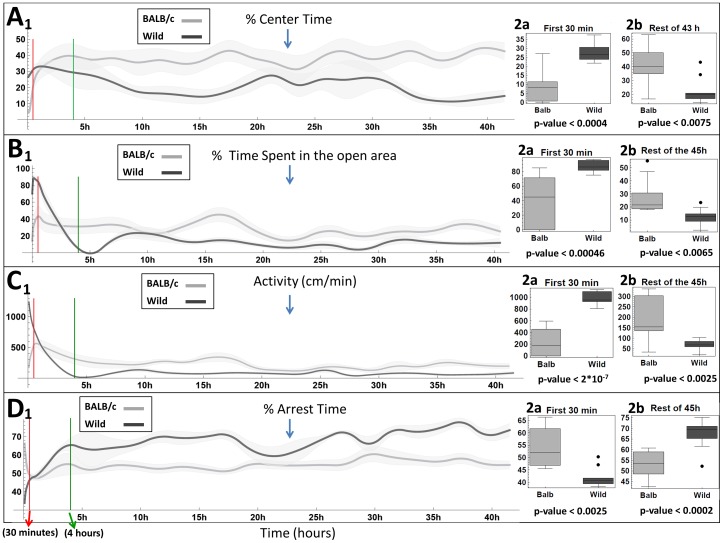
The dynamics of the 4 classical measures of anxiety depicted in**figure 1** are plotted here for the two strains along a 45 h period. Note the relative stability over the period above 4 h (green line) as compared to the first 1/2 h (red line). Box plots (right panels) compare the respective values in the first 1/2 h and the rest of the session. As shown, BALB/c mice score significantly higher over the first 1/2 h, whereas wild mice score significantly higher over the rest of the 45 h session.

Plotting the dynamics of the same measures, averaged over 3 h periods, for a 45 h duration reveals that reversal in the behavior of the 2 strains is stably maintained across the whole testing duration following the first few hours (Figure 2). Taking the first reversal of trend as a sign for the end of the habituation stage we took 4 hours as defining the upper bound of this habituation stage (see Methods section in Information S1). During the first 1/2 h interval the BALB/c mice score significantly lower on the 4 measures, implying higher anxiety than the wild mice, whereas during the long-lasting, relatively stable portion that follows they score significantly higher, implying lower estimated anxiety (for 3 additional variables that support these conclusions see Figure S2).

Since an animal model of Generalized Anxiety Disorder should be designed for the discovery of new drugs that would attenuate *chronic* anxiety, it should correspondingly, by definition, demonstrate long-lasting and stable characteristics of anxious behavior. The fallacy of measuring chronic anxiety at the wrong stage is highlighted by the red vertical 30 minute bar-lines demarcating in the panels of Figures 1, 2 and S1, S2, the maximal interval of the data-collecting stage in currently used tests of anxiolytic drugs (with the exception of studies that took place in home cage environments in which mice were fully habituated, e.g. [35],[36]; in most studies this line extends for an even shorter duration of only the first few minutes where the behavioural differences are even more substantial). These common testing durations are all within the habituation stage demarcated by the green lines in the same figures. The behavior measured during that interval belongs to a short transient, showing a consistent and large change across all measures, apparently reflecting habituation to the setup, and *not* characterizing the behavior of the two strains during the stable stage. Using the test-retest procedure for the evaluation of temporal stability of the behavior as a remedy for the short test duration [17] merely replicates the fallacy by taking the inappropriate measurements twice.

The fallacy of measuring chronic anxiety in the wrong animal-model is evidenced by the wild mice values of anxious behavior during most of the session: lower proportion of time spent in the open area, of activity, of centre time, lower number of transitions (Figures 2, S2), and higher values of the arrest measures, all support the behaviour of the wild progenitor as an appropriate search image for an animal-model of chronic anxiety compared to its domesticated *mus laboratorius* counterpart, whose validity as an animal-model of chronic anxiety is refuted by its calm behavior across the enduring stable stage.

**Figure 3:**
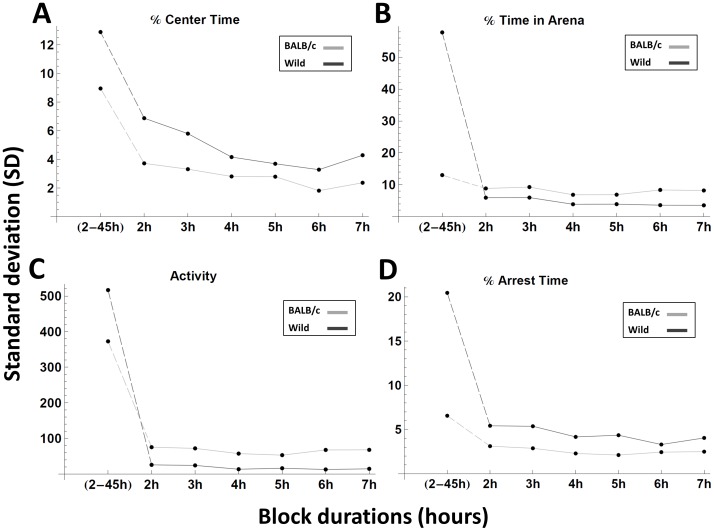
The stability of each of the anxiety measures is estimated by plotting the difference between the mean of the measure’s value in the first two hours and the overall mean for the period extending between 5 h and 45 h (first point in each graph), and then the standard deviation of fixed, non-overlapping blocks of 2 h, 3 h….7 h, all starting at the fifth hour (subsequent points in each graph).

Finally, the fallacy of estimating chronic anxiety on the basis of a short time interval encompassing at most 30 minutes characterized by a consistent and large change across all measures is evident (Figures 1, 2 and S1, S2). Too short sample durations would most likely yield variable and even faulty estimation of the anxiety level. Even at later stages the mice exhibit bouts of short durations with fluctuating behaviour, but when averaged over longer durations they are quite stable. From Figure 3 it can be seen that by 4 hours stability across the non-overlapping periods of time has leveled off for all measures. Further, note in Figure 3 the difference between the mean value of the first 2 hours period and the overall mean of the 5–45 hours interval (leftmost point in each caption), as compared to the variability of the means of the other 2 hours non-overlapping periods relative to same mean, as expressed by the standard deviation (see Figure S3 for stability analysis of the 3 additional measures plotted in Figure S2. See also additional explanation in the ‘stability assessment’ paragraph in the Methods section in Information S1).

## Discussion

Given present knowledge regarding habituation to a new state, large behavioral changes in the first stage are expected, as indeed shown: we have all been measuring a tail, not realizing that it happened to be attached to an elephant.

We have seen that using the classical measures of anxiety for the wild strain over some duration of time starting about 4 hours after first exposure to the open field, seems to capture *chronic* anxiety in the current setting. Of course this relies on the common assumption in the field that these measures indeed reflect anxiety. To fully establish that the behavioral prototype exhibited by wild mice over the stable period is an adequate model of chronic anxiety, it would be necessary to examine in our proposed setup intact and manipulated behavior of wild-derived animal models as has been recently suggested [37]. This can be accomplished by pharmacologists using anxiolytic and anxiogenic drugs [38], by geneticists running genetically engineered mice, and by studying the effects of environmental manipulations that change the animals’ stress level.

Where feasible, drugs could be delivered, long before and all the way through the session, *ad libitum* in the drinking water, recording before the experiment the average water intake per day and preparing solutions so that each strain would consume the required dosages. Drug plasma levels could be determined for the chronically treated mice (e.g., [39]). Drug could also be administered continuously via osmotic minipumps for days until plasma drug levels were found to be within the clinical range (e.g., [40]). A session in the DIEM setup would commence only after required drug plasma levels were achieved and maintained within the clinical range, with a 24 h adaptation period in the home cage followed by an 8, 16, or 24 hours period session of free exploration.

In a way the present paper presents a call for obtaining a wider perspective on the object of measurement, *before* setting out to measure it. From this vantage point, the hypothetical causes for the failure of animal models listed in Table S1 report, as does the present study, the absence of such wider perspective. Steckler et al. [41] report poor separation between state or trait anxiety (our remedy: clear separation between state and trait stages in the model’s behavior). Nestler and Hyman [42] report poor correspondence between human and animal symptoms and disagreement on what counts as a good disease model (our remedy: correspondence between chronic human anxiety and long-term stable stage in model). These researchers also report difficulty in using DSM criteria to construct a mouse model of mental illness (our remedy: again, correspondence between the stable stage in the model and modeled chronic disturbance). Sams-Dodd [43] reports underestimation of complexity. Viewing the object at the proper scale should alleviate some of the problems listed in Table S1.

Our results indicate i) a search for a wild-like animal model that would show anxious behavior during the long-lasting stage. For example, the wild-derived CAST strain that shows similar behaviour to that of the wild progenitor. Since this and other wild derived strains preserve some characteristics of their wild progenitors (e.g., [30],[44]), it is likely that they will also resemble wild *Mus musculus* in exhibiting stable/chronic anxious behavior. Measuring behavior ii) during this stable stage, iii) over a long enough time interval in order to cancel out unavoidable behavioral fluctuations. Starting measurement from the end of the 4^th^ hour and then recording a time interval of 4 hours is sufficiently long for characterising chronic anxiety for all variables (requiring sessions of 8 hours) in the current setting. It should be noted, however, that the sharp boundary (green line) marking the end of the “habituation phase” only estimates the average end of habituation for the few measures used in the present study in two strains. A specific threshold might be estimated for any tested animal/strain based on the data collected in the specific experiment. Habituation to home cage environment was reported to take as much as several days [45]. In our own experiments in the DIEM assay we isolated 12 behavioral landmarks, representing equi-emotional states that appear in much the same order in most mice but differ substantially across animals in the timing of their appearance [26],[27]. Finally, iv) we suggest to replace the statistical summary measurements that might be useful for characterizing the stable period, with a novel measurement methodology for quantifying the build up in extent and complexity of behavior during the drastic and eventful transient that consists of a response to novelty extending over the first few hours [26],[27].

Direct observation of the recorded behaviour reveals that as soon as the whole arena becomes a familiar, heavily-trodden place (100% coverage), the wild mice crouch at the doorway for long time intervals, then dart along the wall, alternating between high speed progression segments and long arrests, avoiding the centre, and performing risk assessment stretch-attends as though having a strong aversion toward the exposed area (Figure S4). Anxious behavior in wild mice is thus the default, perhaps in a similar way to the situation in human generalized anxiety – both are “chronic” in the sense that they are resistant to habituation. While habituation to novelty would be advantageous in a mouse’s home range, it would have been disastrous for the a mouse to become habituated to inhospitable gaps between habitat patches, where small animals run in their familiar home range from patch to patch, slowing down and stopping in the proximity of a shelter or for the sake of foraging [46], making crossing decisions only when the distance of a sheltered detour justifies the cross [47]. In the wild, the response to novelty would thus reflect state anxiety whereas the response to exposed space would reflect generalized “trait” anxiety. Lister’s [8] emphasis on ethological models is corroborated by us: the blueprint is provided by wild mice enduring anxious behavior (see Note S1), where measurements should be taken within a long-enough time window.

In summary, supporting or refuting the validity of the classical measures of anxiety is beyond the scope of the present study. Adopting these measures exposes, however, a problem with the time in the session in which they are measured and with the duration of measurement. Establishing the wild mouse as the prototype animal model for the study of chronic anxiety requires extensive pharmacological work with wild derived strains. Its behavior highlights, however, the inadequacy of current animal models of chronic anxiety. More generally, while focusing on molecular, cellular and genetic mechanisms underlying the behavior of animal models led to remarkable advances, making sense of the implications of these advances for the brain/behavior interface requires a wider perspective in time, in space, and in the natural history of the examined model, as well as a judicious selection of the measurement procedures and parameters that can capture the essence of the behavior.

## Supporting Information

Figure S1 The dynamics of 2 additional measures of anxiety as well as of maximal speed, across the first 5 hours of the sessions of BALB/c inbred mice and wild mice. A: Arrest Duration, B: the Number of Transitions between the home cage and the arena, C: Maximal speed, all averaged over mice in each strain group. Red vertical lines demarcate the end of the first half hour. Note the large change during the presented period especially across the first 1/2 h period, which is the maximal session length used in common studies of anxiety.
(TIF)

Figure S2 The dynamics of 2 additional measures of anxiety as well as of maximal speed across 45 hour sessions of BALB/c inbred mice and wild mice. A: Arrest Duration, B: the number of transitions between the home cage and the arena, C: Maximal speed, all averaged over mice in each strain group. Red vertical lines demarcate the end of the first half hour and green vertical lines demarcate the end of the habituation phase. Box plot summaries (right panel) compare the respective values in the first half hour and the rest of the session (44.5 h).
(TIF)

Figure S3 As in Figure 3, the stability of 3 additional measures is estimated by plotting the difference between the mean of the measure’s value in the first two hours and the overall mean for the period extending between 5 h and 45 h (first point in each graph), and then the standard deviation of fixed, non-overlapping blocks of 2 h, 3 h….7 h, all starting at the fifth hour (subsequent points in each graph).
(TIF)

Figure S4 As soon as the whole arena becomes a familiar, heavily-trodden place (100% coverage; excursions 1–205), the wild mice perform what appears to be anxious behavior: they peep and hide (excursion 213), and perform short (excursions 207, 209 and 212) and long (excursions 206, 208, 210, 211 and 214) excursions along the wall while avoiding the center.
(TIF)

Figure S5 Illustrations of a single excursion in a BALB/c mouse and in a wild mouse, deep into the stable stage (respectively excursions #304 and #125). Blue lines represent the mouse’s paths and red circles represent lingering episodes (staying in place behavior) in the arena, not to be confused with arrests (freezing) in which the speed is 0 [s19],[s20]. The circles’ centers are located at the corresponding lingering location in the arena and their diameters represent lingering durations (see scale in the upper-middle caption). As illustrated, the BALB/c mouse (left) performs extremely long lingering episodes that involve local, low speed, exploratory movements across the exposed area, whereas the wild mouse tends to move and perform relatively short lingering episodes along the wall or near the doorway.
(TIF)

Table S1 Selected hypothetical causes for the failure of animal models to predict the clinical efficacy of drugs.
(DOC)

Note S1 The above poem written by Scotland’s national poet is surely one of the finest poems written by Burns, containing some of the most famous and memorable lines ever written by a poet. It is written in ancient Scotch dialect and it says: Oh you terrified cowardly animal! You do not have to run away so hastily! And you do not have to run about in an undignified way! As I will not run and chase you with a spade and murder you!
(DOC)

Information S1 Supporting text for Figures S1, S2, S3, S4, S5 and Methods.
(DOC)
