# Supplementary figures and images for: Short and Long Term Measures of Anxiety Exhibit Opposite Results
Source: PLoS One. 2012 Oct 31;7(10):e48414. doi: 10.1371/journal.pone.0048414 (PMC3485251; doi:10.1371/journal.pone.0048414)

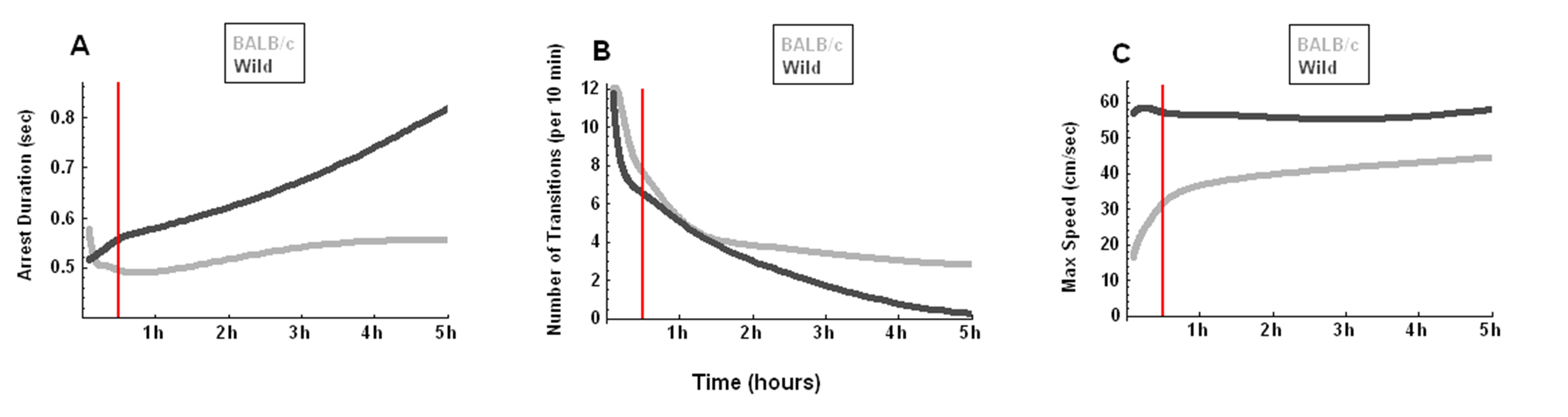

Supplement: Figure S1 — The dynamics of 2 additional measures of anxiety as well as of maximal speed, across the first 5 hours of the sessions of BALB/c inbred mice and wild mice. A: Arrest Duration, B: the Number of Transitions between the home cage and the arena, C: Maximal speed, all averaged over mice in each strain group. Red vertical lines demarcate the end of the first half hour. Note the large change during the presented period especially across the first 1/2 h period, which is the maximal session length used in common studies of anxiety. (TIF) [file pone.0048414.s001.tif]

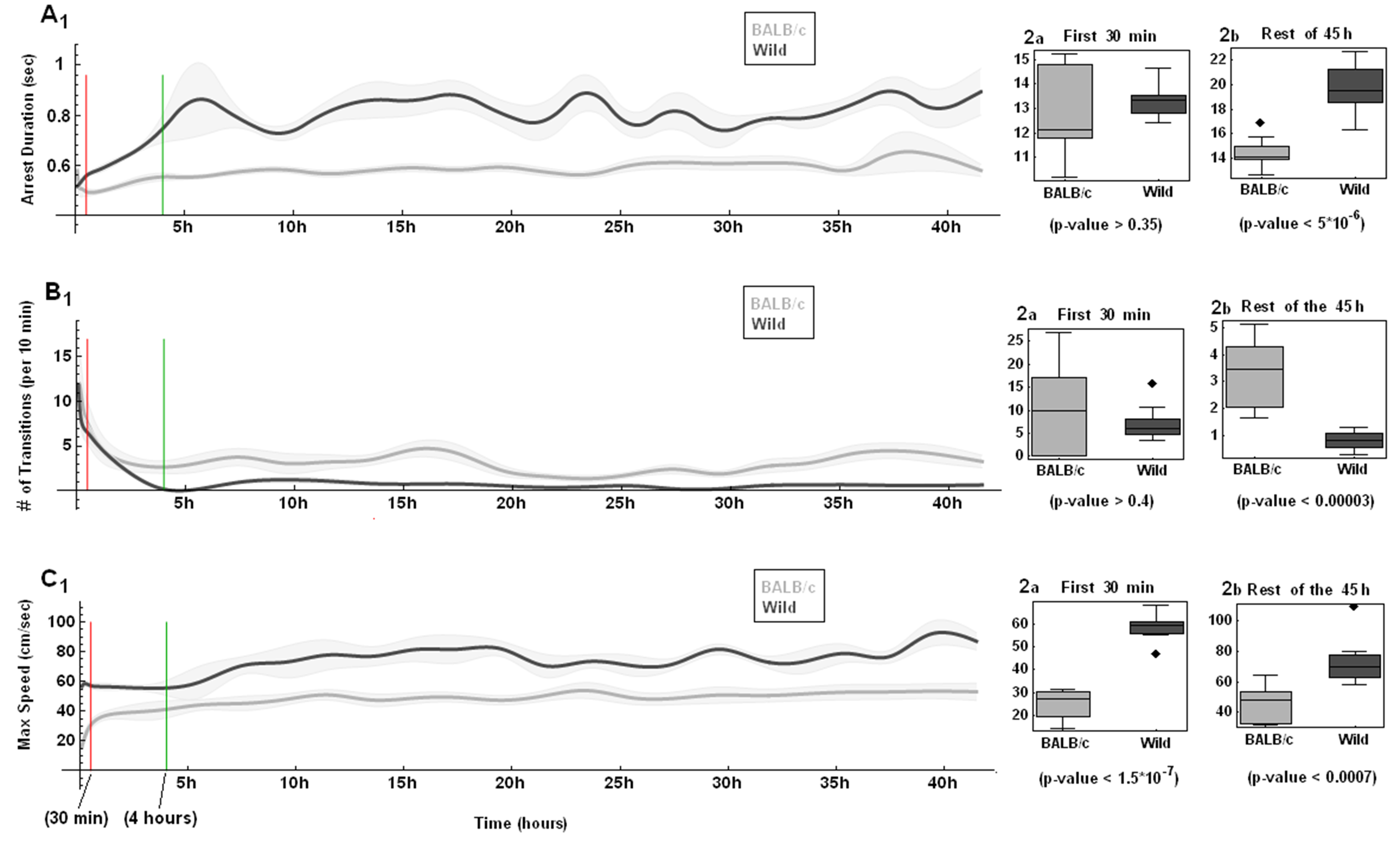

Supplement: Figure S2 — The dynamics of 2 additional measures of anxiety as well as of maximal speed across 45 hour sessions of BALB/c inbred mice and wild mice. A: Arrest Duration, B: the number of transitions between the home cage and the arena, C: Maximal speed, all averaged over mice in each strain group. Red vertical lines demarcate the end of the first half hour and green vertical lines demarcate the end of the habituation phase. Box plot summaries (right panel) compare the respective values in the first half hour and the rest of the session (44.5 h). (TIF) [file pone.0048414.s002.tif]

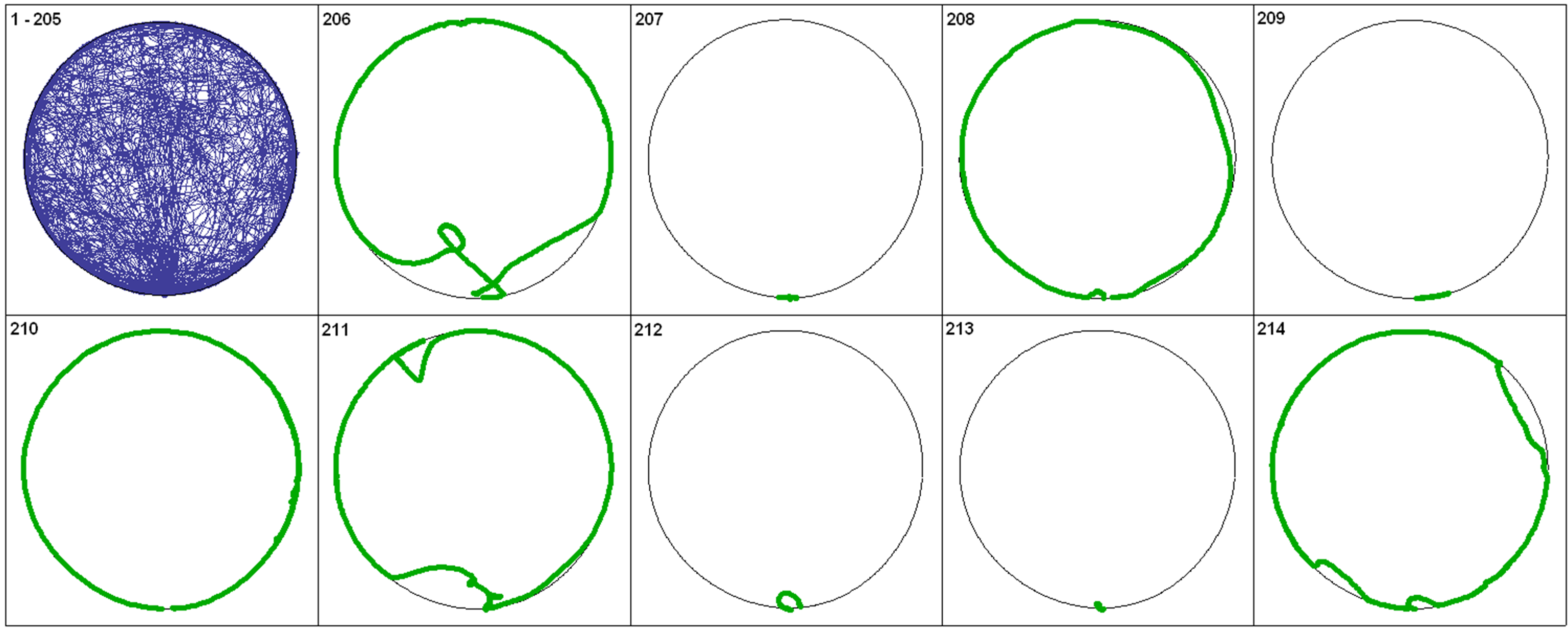

Supplement: Figure S4 — As soon as the whole arena becomes a familiar, heavily-trodden place (100% coverage; excursions 1–205), the wild mice perform what appears to be anxious behavior: they peep and hide (excursion 213), and perform short (excursions 207, 209 and 212) and long (excursions 206, 208, 210, 211 and 214) excursions along the wall while avoiding the center. (TIF) [file pone.0048414.s004.tif]

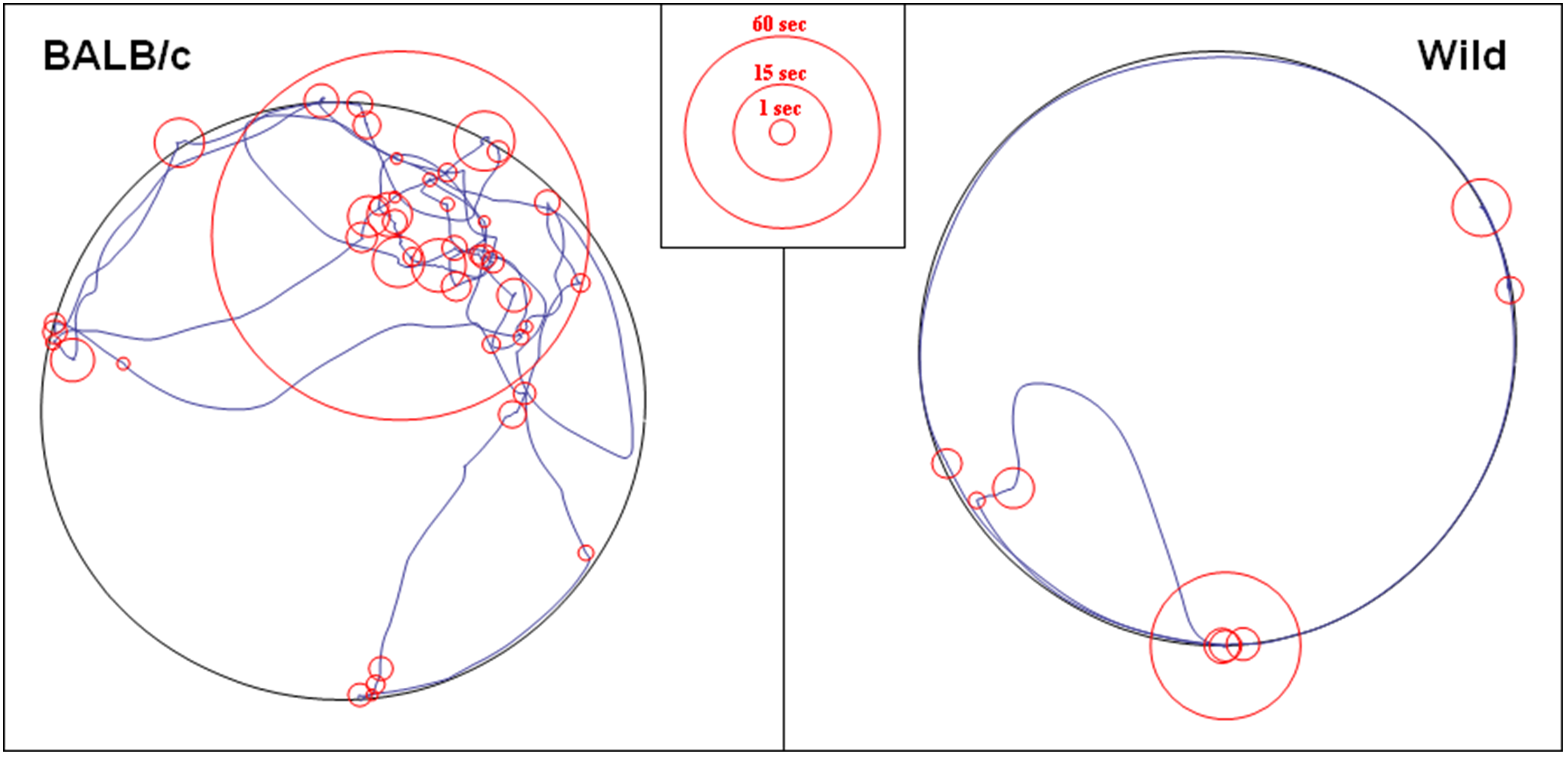

Supplement: Figure S5 — Illustrations of a single excursion in a BALB/c mouse and in a wild mouse, deep into the stable stage (respectively excursions #304 and #125). Blue lines represent the mouse’s paths and red circles represent lingering episodes (staying in place behavior) in the arena, not to be confused with arrests (freezing) in which the speed is 0 [s19],[s20]. The circles’ centers are located at the corresponding lingering location in the arena and their diameters represent lingering durations (see scale in the upper-middle caption). As illustrated, the BALB/c mouse (left) performs extremely long lingering episodes that involve local, low speed, exploratory movements across the exposed area, whereas the wild mouse tends to move and perform relatively short lingering episodes along the wall or near the doorway. (TIF) [file pone.0048414.s005.tif]
