# Supplementary material for: Short and Long Term Measures of Anxiety Exhibit Opposite Results
Source: PLoS One. 2012 Oct 31;7(10):e48414. doi: 10.1371/journal.pone.0048414 (PMC3485251; doi:10.1371/journal.pone.0048414)
Supplement: Table S1 — Selected hypothetical causes for the failure of animal models to predict the clinical efficacy of drugs. (DOC) [file pone.0048414.s006.doc]

***Table S1***

| 1. | Different mouse models of the same disease might have little in common [s15]. | Nestler & Hyman , 2010 |
| --- | --- | --- |
| 2. | Many human symptoms cannot be convincingly ascertained in animals [s15]. | **"** |
| 3. | Disagreement on what counts as a  good disease model as opposed to a tool to investigate the neurobiology of behavior [s15]. | **"** |
| 4. | Difficulties in achieving construct validity of genetic models of mental illness [s15]. | **"** |
| 5. | Difficulty in using DSM criteria to construct a mouse model of mental illness [s15]. | **"** |
| 6. | Deficient flow of information from the clinical/human domain to the preclinical domain [s14]. | Markou et al., 2009, |
| 7. | Insufficient attention to the type of information provided by assays and models used to build the case for clinical evaluation [s14]. | **"** |
| 8. | Insufficient use of truly translational measures in both preclinical and clinical testing [s9]. | Borsini et al, 2002 |
| 9. | Absence of a clear distinction between approach and avoidance dimensions of the behavior [s11]. | Dulawa et al, 1999 |
| 10. | Poor separation between state and trait anxiety [s17]. | Steckler et al, 2008 |
| 11. | Focusing on disease syndromes instead of focusing on dimensions of illness that cut across diseases [s12],[s13]. | Geyer and Markou, 1995; Hyman and Fenton, 2003 |
| 12. | Ignoring alternative explanations for findings, like side effects or main effects in a different domain [s10]. | Bouwknecht & Paylor, 2008 |
| 13. | Tendency to focus narrowly on the genetic or molecular target and to underestimate the complexity of the physiological role of the target in the intact organism [s16]. | Sams-Dodd, 2005 |

**Table S1. Selected hypothetical causes for the failure of animal models to predict the clinical efficacy of drugs.**
