# Supplementary material for: Short and Long Term Measures of Anxiety Exhibit Opposite Results
Source: PLoS One. 2012 Oct 31;7(10):e48414. doi: 10.1371/journal.pone.0048414 (PMC3485251; doi:10.1371/journal.pone.0048414)
Supplement: Note S1 — The above poem written by Scotland’s national poet is surely one of the finest poems written by Burns, containing some of the most famous and memorable lines ever written by a poet. It is written in ancient Scotch dialect and it says: Oh you terrified cowardly animal! You do not have to run away so hastily! And you do not have to run about in an undignified way! As I will not run and chase you with a spade and murder you! (DOC) [file pone.0048414.s007.doc]

The anxious constitution of the wild mouse has been superbly described, as early as 1785, by the memorable lines written by Robert Burns:

* Wee, sleekit, cow'rin, tim'rous beastie,
O, what a panic's in thy breastie!
Thou need na start awa sae hasty,
Wi' bickering brattle!

I wad be laith to rin an' chase thee,

Wi' murd'ring pattle!

*To a Mouse*, *On turning her up in her nest, with the plough*, Robert Burns, 1785.
