# Supplementary material for: Short and Long Term Measures of Anxiety Exhibit Opposite Results
Source: PLoS One. 2012 Oct 31;7(10):e48414. doi: 10.1371/journal.pone.0048414 (PMC3485251; doi:10.1371/journal.pone.0048414)
Supplement: Information S1 — Supporting text for Figures S1, S2, S3, S4, S5 and Methods. (DOC) [file pone.0048414.s008.doc]

**Supporting Information**

***Supporting figures***

Figure S1 presents the dynamics of 2 additional common measures of anxiety as well as the dynamics of maximal speed. The first one is *Arrest Duration*, which represents the median duration of arrest episodes, per fixed time intervals across the session. Arrests or freezing episodes have been manually scored in the past from video, to estimate anxiety in a variety of contexts [s1],[s2],[s3]. Here, using computational tools (see Methods section in the Supporting Information), arrest measures are automatically identified and calculated for the first time for a period of 45 hours. The second is *Number of Transitions* between home cage and arena, which is equivalent to the classical number of transitions in the Light-Dark Box and sometimes in the EPM test [s1]. As shown, these two measures also expose a short transient leading to a long stable stage. During the stable stage, the BALB/c mice score lower on arrest duration implying lower anxiety than the wild mice, and higher on number of transitions, also implying lower anxiety. The inversion between the strains evident in the other measures and in *Arrest Duration*, is absent, however, in the *Number of Transitions* between home cage and arena. Interestingly, the speed of the two strains does not show the inversion characterizing five of the other measures (Fig. S1c).

Figure S2 presents the dynamics of the same 3 variables plotted in figure S1 along a 45h period. As shown, for both *Arrest Duration* and *Number of Transitions*, the values during the last 40h are relatively stable, the wild mice manifesting higher anxiety than the BALB/c mice. Interestingly, the BALB/c mice show consistently lower speed values despite their higher activity level during most of the session (Fig. 2C).

Figure S4 presents the path plots of 214 successive excursions performed by a selected wild mouse. The first 205 excursions are superimposed on top of each other in the leftmost top panel; the rest of the panels display separately the excursions that follow. As illustrated, while traveling over the heavily beaten path, instead of showing habituation to the exposed area, the mouse regresses to center avoidance.

Figure S5 compares the path plots and arrest episodes embedded in one representative excursion for a BALB/c mouse (left) and a wild mouse (right). Arrest durations are represented by red circles centered at the arrest location in the arena and circle diameter represents arrest duration (we use square root transformation for visibility of both short and long arrests).

***Methods***

*Animals.* The wild group included 9 first-generation-in-captivity wild male subjects whose *Mus musculus domesticus* parents were caught in a rural environment in Israel (Kfar Malal). The BALB/c mice included 12 males purchased from Harlan laboratories Israel. All subjects were kept in a 12:12 light cycle (Light: 06:00-18:00) at 22°c room temperature with water and food *ad libitum*, maintained in facilities fully accredited by NIH Animal Welfare Assurance Number A5010-01 (TAU) and tested at 10-11 weeks of age. The male wild mice were separated from their family when they were 3 weeks of age and housed in new cages (group size depended on the number of male mice in each litter and ranged between 1 to 3 mice per group). The BALB/c mice were singly housed due to their known inter-male aggressiveness. The studies were conducted in accordance with the Guide for Care and Use of Laboratory Animals provided by the NIH, ‘Principles of Laboratory Animal Care’ (NIH publication no. 86–23, 1996). To rule out the possibility that *the Corpus Callosum* was absent in some of the BALB/c mice, all the mice belonging to this strain were screened by MRI and found to have a normal structure. All mice were screened while being anesthetized with Isoflurane during the whole procedure and woke up immediately after the test

*Experimental setup.*

The Dimensionality Emergence assay (DIEM) setup that was used in this study is inspired by the free exploration paradigm [s4],[s5] and was described in details in a previous paper [s6]. This assay consists of a 250cm diameter circular arena having a non-porous gray floor illuminated with an IR projector (880nm) and dim white light (<1 Lux) placed on the ceiling above arena centre, simulating moonlight. The arena is surrounded by a 60cm high, primer gray continuous wall with a single 4X5cm’ doorway leading to an infra-red lit Plexiglas home-cage (30x40x50cm) containing wood- or paper-shavings from the original home cage and food and water *ad lib*. A small Plexiglas box attached to the home cage doorway on its inner side forces the mouse to pass through it on its way into the arena without carrying along shavings that might distract the tracking system. Arena floor and Plexiglas-box floor are levelled. The vertical home cage wall is firmly attached to the vertical arena wall securing an immediate passage between the arena and the home cage interior (no corridor). 4 heavy curtains separate the arena from the rest of the room. The arena was thoroughly rinsed with water and soap and then dried, and the home cage was replaced by a clean home cage, at the end of each mouse-session.

*Testing Protocol and Analysis.*

Each mouse was housed in a cage attached to the arena with shavings taken from its original home cage for a 24h adjustment period. To increase the likelihood that the mouse’s activity was elicited by the exposure to the novel open space rather than by the diurnal cycle, the session commenced at 10:00 AM - four hours after the onset of the light cycle, which is the non-active part of the cycle of mice, when the doorway barrier was gently removed and kept open throughout the session. The infra red and dim light above the arena were switched on when the mouse was introduced into the home cage (24h prior to door removal). Each session extended over a 45h period. The animals’ location was tracked using EthoVision™ (25 frames per second, 1 pixel=1cm), and smoothed [s7] and segmented using SEE, a software-based Strategy for Exploring Exploration [s8] available at [http://www.tau.ac.il/~ilan99/see/help](http://www.tau.ac.il/_ilan99/see/help). Further analysis was done using Mathematica™.

*Definition of arrests (freezing behaviour)*

To isolate arrests we use the simplest robust smoother - the Running Median

(RM) [s18]. In this procedure each data point in the speed time series is replaced with the median of its neighbouring observations within a sliding window. RM is usually used in an iterated manner called the Repeated Running Median (RRM) [s18]: first smooth the measured data with a RM, next smooth the resultant smoothed series with another RM, possibly with a different window width, and so forth. Such repetitions have an effect of giving more weight to locations closer in time to the center of the window. A proper choice of parameters (the window widths of the Repeated Running Medians) was found to yield identification of arrests that coincides very well with the identification by several experienced observers that examined each arrest several times in the videotape. The choice of the window sizes in the sequence was done by a trial-and-error but also followed Tukey’s guidelines, who recommended repeating the RM steps starting from wider window width to smaller or same size window widths [s18]. Once obtaining a final smoothed set of locations, a run of at least l locations that are not different by more than a small distance ε are marked as arrests. We found that l = 5 (equivalent to 0.2 s at a rate of 25 frames/s) and a very small ε, practically 0, yield an identification of arrests almost identical to that done by experienced human observers. Note, that arrests are not to be confused with lingering episodes: while arrests involve complete freezing or immobility, lingering may consist of arrests interrupted by small motions. For more detail see [s7],[s19].

*Smoothing*

Measures of behavior were smoothed with a symmetric moving average over 3h window that proceeds in steps of 2 hours, except for the very beginning where keeping the symmetry of the window requires shorter duration.

*Duration of the transient habituation phase*

The decision on the end of the transient habituation was based on the first time a reversal point in the smoothed behaviour was reached. Since this depends on the window-width, windows of 0.5h to 1h were used. The habituation thresholds obtained ranged between 1h to 4.5h, depending on the measure, the strain, and the level of smoothing used, and a conservative 4h value was taken.

*Stability assessment*

The stability over x-hours period was assessed by dividing the period after the first 5 hours to non-overlapping periods of duration x and calculating their standard deviation. The minimal requirement of duration needed to assess the behavior over the second, long-lasting period, was derived from the duration at which the stability measure stopped decreasing sharply.

*Box plot summaries*

The box plot representations used in figures 2 and S2 are uniform in their use of the box: the bottom and top of the box are always the 25th and 75th [percentile](http://en.wikipedia.org/wiki/Percentile) (the lower and upper [quartiles](http://en.wikipedia.org/wiki/Quartile), respectively), and the horizontal bar near the middle of the box is always the 50th [percentile](http://en.wikipedia.org/wiki/Percentile) (the [median](http://en.wikipedia.org/wiki/Median)). The ends of the whiskers represent the lowest datum still within 1.5 [Inter Quartile R](http://en.wikipedia.org/wiki/Interquartile_range)ange (IQR) of the lower quartile, and the highest datum still within 1.5 [IQR](http://en.wikipedia.org/wiki/Interquartile_range) of the upper quartile. Outliers are represented as dots either below or above the whiskers' ends.

**References for Supporting Information**

S1. Rodgers RJ, Cole JC (1993) Anxiety enhancement in the murine elevated plus maze by immediate prior exposure to social stressors. Physiol Behav 53: 383-388.

S2. Blanchard DC, Blanchard RJ (2008) Defensive behaviors, fear and anxiety. In R. J. Blanchard, D. C. Blanchard, G. Griebel & D. J. Nutt (Eds. ), Hanbook of anxiety and fear. Amsterdam: Elsevier academic press.

S3. Miyata S, Shimoi T, Hirano S, Yamada N, Hata Y, et al. (2007) Effects of Serotonergic Anxiolytics on the Freezing Behavior in the Elevated Open-Platform Test in Mice. J Pharmacol Sci 105: 272-278.

S4. Welker WI (1957) "Free" versus "forced" exploration of a novel situation by rats. Psychol Rep 3: 95-108.

S5. Hughes RN (1965) Food deprivation and locomotor exploration in the white rat. Anim Behav 13: 30-32.

S6. Fonio E, Benjamini Y, Golani I (2009) Freedom of movement and the stability of its unfolding in free exploration of mice. Proc Natl Acad Sci U S A 106: 21335-21340.

S7. Hen I, Sakov A, Kafkafi N, Golani I, Benjamini Y (2004) The dynamics of spatial behavior: how can robust smoothing techniques help? J Neurosci Methods 133: 161-172.

S8. Drai D, Golani I (2001) SEE: a tool for the visualization and analysis of rodent exploratory behavior. Neurosci Biobehav Rev 25: 409-426.

S9. Borsini F, Podhorna J, Marazziti D (2002) Do animal models of anxiety predict anxiolytic-like effects of antidepressants? Psychopharmacology (Berl) 163: 121-141.

S10. Bouwknecht JA, Paylor R (2008) Pitfalls in the interpretation of genetic and pharmacological effects on anxiety-like behaviour in rodents. Behav Pharmacol 19: 385-402.

S11. Dulawa SC, Grandy DK, Low MJ, Paulus MP, Geyer MA (1999). Dopamine D4 receptor-knock-out mice exhibit reduced exploration of novel stimuli. J Neurosci 19: 9550-9556.

S12. Geyer MA, Markou A (1995). Animal models of psychiatric disorders. In: Bloom FE, Kupfer DJ (eds). Psychopharmacology: The Fourth Generation of Progress. Raven Press: New York. pp 787-798.

S13. Hyman SE, Fenton WS (2003) Medicine: what are the right targets for psychopharmacology? Science 299: 350-351.

S14. Markou A, Chiamulera C, Geyer MA, Tricklebank M, Steckler T (2009) Removing obstacle s in neuroscience drug discovery: the future path for animal models. Neuropsychopharmacology 34: 74-89.

S15. Nestler EJ, Hyman SE (2010) Animal models of neuropsychiatric Disorders. Nat neurosci 13: 1161-1169.

S16. Sams-Dodd F (2005) Target-based drug discovery: is something wrong? [Drug Discov Today](http://www.sciencedirect.com/science/journal/13596446) [10](http://www.sciencedirect.com/science?_ob=PublicationURL&_tockey=%23TOC%235020%232005%23999899997%23572466%23FLA%23&_cdi=5020&_pubType=J&view=c&_auth=y&_acct=C000005078&_version=1&_urlVersion=0&_userid=48161&md5=5611cae7bf709cae72fcbe7610e11da7): 139-147.

S17. Steckler T, Stein M, Holmes A (2008). Developing novel anxiolytics: improving preclinical detection and clinical assessment. In: McArthur RA, Borsini F (eds). Animal and Translational Models in Psychiatric Disorders, Vol. 1: Psychiatric Disorders. Academic Press, London. pp 117-132.

S18. Tukey JW (1977). Exploratory data analysis, Addison-Wesley Publishing Company, Reading, Massachusetts.

S19. Benjamini Y, Lipkind D, Horev G, Fonio E, Kafkafi N, et al. (2010). Ten ways to improve the quality of descriptions of whole-animal movement. Neurosci Biobehav Rev 34: 1351-1365.

S20. Drai D, Benjamini Y, Golani I (2000) Statistical discrimination of natural modes of motion in rat exploratory behavior. J Neurosci Methods 96: 119-131.
